# Lower circulating insulin-like growth factor-I is associated with better cognition in females with exceptional Longevity without compromise to muscle mass and function

**DOI:** 10.18632/aging.101063

**Published:** 2016-10-14

**Authors:** Leland Perice, Nir Barzilai, Joe Verghese, Erica F. Weiss, Roee Holtzer, Pinchas Cohen, Sofiya Milman

**Affiliations:** ^1^ Albert Einstein College of Medicine, Bronx, NY 10461, USA; ^2^ Department of Medicine, Institute for Aging Research, Albert Einstein College of Medicine, Bronx, NY 10461, USA; ^3^ Department of Genetics, Albert Einstein College of Medicine, Bronx, NY 10461, USA; ^4^ Department of Neurology, Albert Einstein College of Medicine, Bronx, NY 10461, USA; ^5^ Ferkauf Graduate School of Psychology, Yeshiva University, Bronx, NY 10461; ^6^ Davis School of Gerontology, University of Southern California, Los Angeles, CA 90089, USA

**Keywords:** IGF-I, insulin-like growth factor-1, cognition, exceptional longevity, females

## Abstract

Mutations that reduce somatotropic signaling result in improved lifespan and health-span in model organisms and humans. However, whether reduced circulating insulin-like growth factor-I (IGF-I) level is detrimental to cognitive and muscle function in older adults remains understudied. A cross-sectional analysis was performed in Ashkenazi Jews with exceptional longevity (age ≥95 years). Cognition was assessed using the Mini-Mental State Examination and muscle function with the chair rise test, grip-strength, and gait speed. Muscle mass was estimated using the skeletal muscle index. Serum IGF-I was measured with liquid chromatography mass spectrometry. In gender stratified age-adjusted logistic regression analysis, females with IGF-I levels in the first tertile had lower odds of being cognitively impaired compared to females with IGF-I levels within the upper two tertiles, OR (95% CI) 0.39 (0.19-0.82). The result remained significant after adjustment for multiple parameters. No significant association was identified in males between IGF-I and cognition. No relationship was found between IGF-I tertiles and muscle function and muscle mass in females or males. Lower circulating IGF-I is associated with better cognitive function in females with exceptional longevity, with no detriment to skeletal muscle mass and function.

## INTRODUCTION

Cognitive decline is a highly prevalent condition among the aging population that causes significant morbidity in the elderly and results in rising expense for the healthcare system [1]. Although aging is a major risk factor for cognitive impairment, some individuals with exceptional longevity demonstrate delayed onset of dementia by as much as 13 years, with many not manifesting it at all [2, 3]. The fact that individuals with exceptional longevity possess factors that allow them to delay or avoid age related diseases make them a particularly attractive model for the study of healthy aging [4]. One of the features identified in individuals with exceptional longevity was partial resistance to insulin-like growth factor-1 (IGF-I) resulting from a mutation in the IGF-I receptor gene [5, 6]. Subsequent studies have shown that lower IGF-I and IGF-I/IGF binding protein-3 (IGFBP-3) ratio are associated with extended survival in nonagenarians [7, 8] and better performance at activities of daily living [8].

Despite evidence from humans and experimental models that reduced circulating IGF-I may promote longevity and healthy lifespan [5, 7–13], the role of peripheral IGF-I in cognition and muscle function remains unresolved [14–16]. Several cross-sectional and prospective studies linked lower IGF-I to poorer cognitive function, as well as higher risk for mild cognitive impairment and Alzheimer's disease [17–19]. On the other hand, a recent prospective study in older men associated IGF-I levels in the lowest quintile with less cognitive decline [20]. Adding to this debate are the differences observed between the sexes. For example, in the Rancho-Bernardo cohort higher IGF-I was associated with better cognitive function only among men, but not women [19]. The aging process is also associated with loss of muscle mass and function [21]. Since growth hormone (GH), a dominant stimulant for the peripheral IGF-I production, and circulating IGF-I levels decrease with age [22], their decline has been viewed by some as a cause of aging, as well as age-related cognitive and physical deterioration, with several studies attempting to reverse the effects of aging by replacing GH and IGF-I in humans [23, 24]. Although repletion of GH/IGF-I has been shown to improve muscle mass in older men, these attempts have not been met with success in terms of their effects on muscle function and memory [23, 24].

With the understanding that healthspan extension is an important determinant of healthy aging, we set out to test the hypothesis that individuals with exceptional longevity and low circulating IGF-I levels not only exhibit extended survival, but are also healthier in cognitive and muscle function domains. Furthermore, given our prior findings that low IGF-I benefited females preferentially [7], we tested whether this association is sex-specific in relation to these other clinical outcomes.

## RESULTS

IGF-I levels and cognitive assessment were available for 203 participants, 163 female and 40 male, median age (interquartile range) 97.2 (95.1-99.9) years and 97.5 (95.5-98.8) years, respectively. Measured levels of IGF-I were not found to be significantly different between males and females; however, the IGF-I/IGFBP-3 ratio was significantly lower in females compared to males, mean ± standard deviation (SD) 0.11±0.03 vs. 0.13±0.04, respectively, p<0.01 (Table 1).

**Table 1:** Measures of IGF-I, IGFBPs, and IGF-I/IGFBP-3 molar ratios in females and males

Measurement	Females (n=163)	Males (n=40)	p-value
**Age** (yrs)	97.2 (95.1-99.9)	97.5 (95.5-98.8)	>0.99
**IGF-I**, ng/mL (n=203)	99±42	87±32	0.10
**IGFBP-1**, ng/mL (n=195)	16 (0-27)	18 (0-26)	0.93
**IGFBP-3**, mg/mL (n=201)	3.3±1.0	2.6±0.8	<0.01
**IGF-I/IGFBP-3 molar ratio** (n=201)	0.11±0.03	0.13±0.04	<0.01

**Figure 1:**
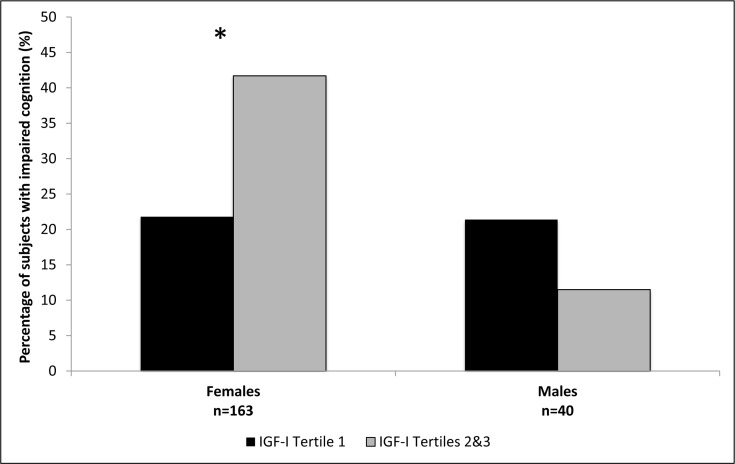
Percentage of subjects with impaired cognition according to IGF-I tertiles, in females and males. *Females p=0.01; Males p=0.65.

The female and male cohorts were separated into tertiles based on IGF-I levels, with the second and third tertiles combined due to similar associations with cognition. Participant characteristics for females and males are shown in Tables 2 and 3, respectively. In both females and males mean IGF-I levels, IGFBP-3 levels, and IGF-I/IGFBP-3 molar ratios were significantly lower in the first tertile compared to the combined upper two tertiles. In the group with IGF-I levels within the combined upper tertiles 41.7% of females demonstrated cognitive impairment on MMSE compared to 21.8% in the lowest IGF-I tertile, p=0.01 (Figure 1). No statistically significant difference in cognitive function on MMSE based on IGF-I tertiles was observed among the males, p=0.65 (Figure 1). The IGF-I groups did not significantly differ by age, height, BMI, education, depression or prevalence of disease in the female or male cohorts.

**Table 2:** Female characteristics according to IGF-I tertiles

Female Characteristics	IGF-I Tertile 1(n=55)	IGF-I Tertiles 2 and 3 (n=108)	p-value
**Age**, years	97.3 (96.3-99.0)	97.1(95.9-100.3)	0.97
**Height**, m (n=131)	1.59±0.05	1.59±0.06	0.41
**BMI,** kg/m^2^ (n=128)	21.0±3.0	21.6±3.3	0.33
**IGF-I**, ng/mL	58±13(range 25-75)	119±35(range 76-268)	<0.01
**IGFBP-1**, ng/mL (n=155)	17 (5-33)	15.5 (0-26)	0.15
**IGFBP-3**, mg/mL, (n=161)	2.6±0.7	3.7±0.9	<0.01
**IGF-I/IGFBP-3 molar ratio**, (n=161)	0.09±0.03	0.12±0.03	<0.01
**Glucose**, mg/dL, (n=145)	99±35	107±47	0.34
**25(OH)_2_ vitamin D**, mg/dL, (n=97)	26.8±11.9	26.5±10.2	0.89
MMSE score* (n=123)	29 (8-30)	27 (2-30)	0.04
**Hypertension**, % (n=153)	54.7	60.0	0.53
**Cardiovascular disease,** % (n=155)	24.1	28.7	0.54
**Diabetes mellitus**, % (n=155)	9.3	8.9	0.94
**Cancer**, % (n=153)	28.9	15.8	0.06
**Education**, years (n=133)	13.6±3.4	12.9±3.1	0.22
**Depression** (%), (n=115)	35.0	44.0	0.35
**Tobacco use, ever** (%), (n=144)	46.9	25.3	<0.01
**Alcohol use at age 70 yrs** (%), (n=139)	57.5	44.6	0.15

* Median (IQR) scores for MMSE exclude subjects who were scored using the blind MMSE.

**Table 3:** Male characteristics according to IGF-I tertiles

Male Characteristics	IGF-I Tertile 1 (n=14)	IGF-I Tertiles 2 and 3 (n=26)	p-value
**Age**, years	98 (96.2-99.4)	97.2 (96.3-100)	0.57
**Height**, m (n=35)	1.69±0.08	1.73±0.08	0.26
**BMI**, kg/m^2^ (n=33)	20.3±2.0	21.9±2.4	0.09
**IGF-I**, ng/mL	55±8 (range 36-65)	104±26 (range 69-164)	<0.01
**IGFBP-1**, ng/mL	22 (10-54)	14 (0-25)	0.23
**IGFBP-3**, mg/mL	2.2 ±0.8	2.8±0.8	0.02
**IGF-I/IGFBP-3 molar ratio**	0.10±0.05	0.13±0.04	0.01
**Glucose**, mg/dL, (n=38)	98±24	101±30	0.81
**25(OH)2 vitamin D**, mg/dL, (n=18)	20.7±9.2	29.5±11.1	0.17
**MMSE** score* (n=34)	27.5 (24-30)	29 (25-30)	0.24
**Hypertension**, % (n=39)	35.7	44.0	0.61
**Cardiovascular disease**, % (n=37)	46.2	37.5	0.61
**Diabetes mellitus**, % (n=37)	7.7	16.7	0.64
**Cancer**, % (n=40)	28.6	7.7	0.16
**Education**, years (n=35)	14.3±5.8	15.7±6.3	0.51
**Depression** (%), (n=33)	33.3	14.3	0.38
**Tobacco use, ever** (%), (n=35)	33.3	52.2	0.29
**Alcohol use at age 70 yrs** (%), (n=35)	91.7	78.3	0.64

* Median (IQR) scores for MMSE exclude subjects who were scored using the blind MMSE.

The females in the first IGF-I tertile were found to have significantly lower odds of cognitive impairment compared to combined upper two tertiles, OR (95% CI) 0.39 (0.19-0.82), Table 4. This result remained signi-ficant after adjustment for age, OR 0.39 (0.19-0.83), as well as after additional adjustment for diabetes mellitus, hypertension, cancer, and cardiovascular disease, OR 0.42 (0.19-0.97). The model adjusted for age, depression, education, history of tobacco use and alcohol use, and IGFBP-3 level was also significant (n=104 for the adjusted model), OR 0.24 (0.06-0.98). No significant association was noted between IGF-I and cognition in males, OR 2.09 (0.36-12.08) for the unadjusted model and after adjustment for age, OR 2.47 (0.40-15.09), Table 4.

**Table 4:** Odds ratios (OR) for cognitive impairment according to IGF-I tertiles in females and males

Model	IGF-I Tertile 1 OR	IGF-I Tertiles 2 and 3 Reference OR	p-value
**Females**			
	**n=55**	**n=108**	
**Model A: Unadjusted (n=163)**	0.39 (0.19-0.82)	1	0.01
**Model B: Adjusted for age (n=163)**	0.39 (0.19-0.83)	1	0.01
**Model C: Adjusted for age, diabetes, hypertension, cancer, cardiovascular disease (n=148)**	0.42 (0.19-0.97)	1	0.04
**Model D: Adjusted for age, depression, education, tobacco use, alcohol use, and IGFBP-3 (n=104)**	0.24 (0.06-0.98)	1	0.047
**Males**			
	**n=14**	**n=26**	
**Model A: Unadjusted (n=40)**	2.09 (0.36-12.08)	1	p=0.41
**Model B: Adjusted for age (n=40)**	2.47 (0.40-15.09)	1	p=0.33

Table 5 shows the results for muscle mass and function. The percentage of participants with sarcopenia or impaired muscle function, assessed with the chair rise test, grip strength, and gait speed, did not differ significantly between the lowest tertile of IGF-I and the combined upper two tertiles of IGF-I in women or men. There was also no significant association between the IGF-I tertiles and the presence of frailty in both sexes (Table 5).

**Table 5:** Skeletal muscle mass and muscle functional assessment according to IGF-I tertiles

Skeletal muscle mass and functional assessment	IGF-I Tertile 1	IGF-I Tertiles 2 and 3	p-value
**Females**			
**Sarcopenia (n=72), %**	18.2	28.0	0.56
**Failed chair rise test (n=51), %**	81.0	80.0	0.93
**Weak grip strength (n=52), %**	50.0	43.3	0.63
**Slow gait (n=40), %**	47.1	52.2	0.75
**Frailty (n=42), %**	76.5	64.0	0.51
**Males**			
**Sarcopenia (n=15), %**	21.4	11.5	0.65
**Failed chair rise test (n=24), %**	75.0	54.6	0.60
**Weak grip strength (n=24), %**	66.7	33.3	0.21
**Slow gait (n=19), %**	57.1	33.3	0.38
**Frailty (n=19), %**	50.0	46.2	>0.99

## DISCUSSION

In this cross-sectional analysis lower serum IGF-I levels were found to be associated with better cognitive function on the MMSE in females with exceptional longevity, but not in males. Furthermore, no detriment to muscle mass or function was observed in this cohort among women or men with IGF-I levels within the lowest tertile of IGF-I compared to individuals with IGF-I in the upper two tertiles. Our study is the first to demonstrate a gender specific negative association between IGF-I and cognition in the extremely elderly. This supports previous data showing that lower IGF-I may have protective effects in aging [8, 25] that may be gender specific [5, 19, 26].

Although findings from several other groups differed from ours, there were substantial differences between the studied cohorts and study design that may explain the varied results. For instance, the Rancho Bernardo study did not find an association between MMSE scores and IGF-I levels among females with a median age 74 years [19]. On the other hand, a prospective study by Okereke et al. noted that higher IGF-I/IGFBP-3 ratio was associated with better cognition, as measured by a telephone version of the MMSE, after 10 years of follow-up among females aged 60-68 at baseline [18]. Several factors should be considered when interpreting these results in the context of our findings. First, it is important to recognize that the Rancho Bernardo cohort included relatively healthy females, who performed very well on the MMSE overall, thus suggesting that the narrow distribution of MMSE scores among the tertiles of IGF-I may have led to a negative result [19]. Second, circulating IGF-I may exert a protective effect on cognition during the 7^th^ and 8th decade of life, but be detrimental in older age groups. Circulating GH/IGF-I levels normally peak in the 2nd decade of life and then decline until they plateau around the 6th decade of life [27]. It is presently unknown at what age an attenuation of GH and/or IGF-I results in a protective effect on cognition; however, protection may be a hallmark of older age. On the other hand, if IGF-I level declines too precipitously, it may reflect premature aging.

The observed difference in age-dependent effect of IGF-I highlights the importance for further investigation into the age specific aspects of the GH/IGF-I axis.

Although we did not find a significant association between IGF-I and cognition in males, our analysis may have been limited by low power. A recent prospective analysis in men showed less cognitive decline in those with IGF-I levels in lower quintiles compared to men with IGF-I in the top quintile [20]. Furthermore, in men older than 60 years higher IGF-I was associated with lower cognitive processing ability at baseline [20]. Alternatively, in a separate cross sectional cohort of older males, better cognition was noted in men with higher circulating IGF-I levels, a finding suggested by our data as well [19]. Thus, in addition to age, sex-differences need to be considered in investigations of GH/IGF-I as its effects may differ in men and women.

IGF-I signaling contributes to normal brain homeostasis and development [28, 29]. IGF-I is produced both locally in the brain and crosses the blood brain barrier from the peripheral circulation [30–32]. In humans, loss of function mutations in the GH or its receptor result in low circulating levels of IGF-I. Although these mutations clinically manifest with growth failure, delayed skeletal maturation and metabolic problems, notably most have been found to not result in significant cognitive defects [33]. This is unlike mutations in the *IGF-I* gene, which have been documented to result in neurocognitive defects and microcephaly [34]. One mechanism that could potentially explain this difference is that in a state of deficient GH signaling and low peripheral IGF-I, the IGF-I level within the central nervous system may be maintained through autocrine production. Many tissues, including the hypothalamus and brain stem, produce IGF-I that acts via autocrine and paracrine signaling [35]. Furthermore, the Ames dwarf mice that lack anterior pituitary hormones, including GH, have been noted to maintain IGF-I levels and IGF-I activity within the brain [36].

No association was found between tertiles of circulating IGF-I and muscle mass or muscle function in our cohorts. These results suggest that circulating IGF-I plays a minimal role in maintaining muscle mass or strength in individuals with exceptional longevity. It has been shown that mechanical strain can induce skeletal muscle hypertrophy independently of IGF-I in both mouse models with IGF-I and IGF-I receptor mutations [16, 37, 38]. It is possible that intrinsic tension-sensing mechanisms independent of hormones mediate muscle hypertrophy and maintenance [39]. Another potential explanation is that the circulating form of IGF-I analyzed here is unrelated to muscle homeostasis in older women. This was also suggested by a study by Friedlander et al., which found no benefit of a year long IGF-I administration on body composition in post-menopausal women [23]. While it is established that IGF-I is critical for muscle development and adolescent growth [34, 40, 41], its role in muscle maintenance in the elderly is unclear.

This study has several limitations. It used a self-reported medical and social history, creating a potential reporting bias, particularly in the cognitively impaired. Yet, for individuals with exceptional longevity, lifestyle factors might not be as important as genetic factors [42]. Additionally, it is highly unlikely that differential reporting existed given that the reported rates of the many lifestyle parameters and diseases were no different among the groups. In addition, the Ashkenazi Jewish population in the US is largely homogenous in its socioeconomic factors, which limits some potential sources of bias. Another limitation is the small size of our male cohort. While this limits the conclusions that we can make about the male cohort, the results highlight possible differences in IGF-I physiology between men and women, warranting further investigation into the sex-associated effects of IGF-I. Also, given the cross-sectional nature of this analysis it remains unknown whether the subjects had life-long low levels of IGF-I or experienced a decline in IGF-I later in life.

These results suggest that the impact of IGF-I on cognition in older adults is not as straightforward as previously thought and that lower IGF-I may in fact be beneficial to cognition. Individuals with exceptional longevity are a unique group who can serve as a model of healthy aging. The biology learned from these subjects can lead to better understanding of hormonal regulation of aging and disease. More investigation is needed in order to elucidate the sex and age-specific functions of the GH/IGF-I axis before possible therapeutic modulation of the pathway can be explored.

## METHODS

### Study participants

Individuals age 95 years and older of Ashkenazi Jewish descent were recruited from the Northeastern United States for the Longevity Genes Project at Albert Einstein College of Medicine beginning in 1998, as previously described [43]. Study participants were required to have been living independently at age 95, as a reflection of general good health, even if at a later age they developed dependency. Age was verified with government issued identification. A study nurse visited the participants at their residence and performed the study evaluations, including measurements of weight and height. Detailed medical and social histories were obtained using a standardized questionnaire. A venous blood sample was collected and the processed serum was stored at −80°C. Sufficient sample volume was available for 304 individuals. Written informed consent was obtained from the participants or their proxies, in the event that the subject lacked capacity. The study was approved by the Institutional Review Board at the Albert Einstein College of Medicine.

### Cognitive and mood assessment

Cognition was evaluated with the Mini-Mental State Examination (MMSE)[44]. The MMSE was scored out of 30 points for participants without major visual impairment, with a score of ≥25 being representative of normal cognition [45]. A Blind MMSE [46, 47] was utilized for 45 (22%) of participants with severe visual impairment, on which items requiring image processing were excluded. The Blind MMSE was scored out of 22 points [46, 47] and a score of ≥16 indicated normal cognition. Only one subject did not complete the MMSE due to severe dementia and was included in the group with impaired cognition. Although the MMSE may not be a very sensitive tool to pick up mild cognitive changes, it has proven to be a useful asses-sment of cognition in our population with exceptional longevity, in part due to a wide distribution of MMSE scores observed in this cohort [48–50]. Mood was evaluated with a fifteen item Geriatric Depression Scale (GDS), with a score of ≥6 used as a cut score suggestive of depression [51].

### Skeletal muscle mass and function assessment

Skeletal muscle mass was estimated according to the following validated formula: muscle mass (kg)=[(Height^2^/R x 0.401) + (gender x 3.825) + (age x − 0.071)] + 5.102, where height is in centimeters; R is resistance in ohms based on bioimpedance analysis (BIA); for gender men=1 and women=0; age is in years [52]. BIA was measured using Tanita Body Compo-sition Analyzer TBF-300A (Tokyo, Japan). Skeletal muscle index (SMI) was calculated as SMI=muscle mass (kg)/height (m)^2^ and provided normalization of muscle mass for height [53]. SMI of ≤6.75 kg/m^2^ and ≤ 10.75 kg/m^2^ were defined as severe sarcopenia in women and men, respectively, based on associated high physical disability risk in older adults, as identified in the older population sample from the National Health and Nutrition Examination Survey III (NHANES III) [53, 54].

Assessments of muscle function were introduced into the study protocol at a later time (2007), therefore fewer subjects completed these tests. Grip strength was assessed with the use of dynamometer. The dynamo-meter was squeezed with the dominant hand and the maximum grip strength (kg) recorded out of three attempts was used for analysis. Weak grip strength was defined as either a reading that was 1 standard deviation (SD) below sex-specific means or inability to squeeze the dynamometer. Gait speed (m/sec) was calculated based on the time it took to walk a distance of 10 feet (3.048 m). Slow gait speed was defined as gait speed in the lowest quartile, stratified by sex and median height [55]. Subjects who were unable to complete the walking test due to physical limitations were also characterized as having slow gait. Individuals met criteria for frailty if they demonstrated three or more of the following deficits: 1. slow gait speed; 2. weak grip strength; 3. response of “No” to a question on the GDS that asks, “Do you feel full of energy?”; 4. report of less activity in the past 12 months; 5. body mass index (BMI), calculated as weight (kg)/height (m)^2^, in the lowest quintile [56, 57]. BMI was calculated based on reported maximum adult height. Deficit in performing the chair rise test was defined as inability to rise from a chair 5 times without using one's arms for support [58].

### Laboratory measures

IGF-I level was measured in stored serum with liquid chromatography/ mass spectrometry (LC-MS) analysis at Quest Diagnostics Nichols Institute laboratories, San Juan Capistrano, CA. For IGF-I assay the limit of quantification (LOQ) was 15.6 ng/mL and the coefficient of variance (CV) was 3.3% for low control (mean 57.2ng/mL), 3.1% for the medium control (mean 248 ng/mL), 2.8% for the high control (mean 447.1 ng/mL) and 5% for the in-house control (mean 104 ng/mL). IGFBP-1 and IGFBP-3 levels were measured at Quest Diagnostics with chemiluminescent immuno-metric assay (Siemens Immulite 2000). For IGFBP-1 the LOQ was 5 ng/mL and CV is 9.3% for the low control (mean 19.2 ng/mL), 10.1% for the medium control (mean 53.5 ng/mL) and 8.5% for the high control (mean 111.3 ng/mL). For IGFBP-3 the LOQ was 0.5 mg/L and CV was 5.1% for the low control (mean 0.9 mg/L) and 6.1% for the high control (mean 3.56 mg/L) and 6.5% for in-house control. Serum 25-hydroxyvitamin D (25(OH)D) levels were measured by liquid chromatography/ tandem mass spectrometry (LC/MS/MS) analysis at Montefiore Medical Center, Bronx, NY.

### Statistical analysis

Statistical analysis was performed using STATA software, version 12 (StataCorp LP, College Station, TX). Bivariate statistics were used for comparison of baseline characteristics, with non-parametric tests used when appropriate. Normality was assessed by observation of the histograms. Proportions were compared using the Chi square statistic, with Fisher's exact test used when indicated. Sex-stratified multivariable logistic regression models were built, utilizing a manual forward building process, to determine the independent association between IGF-I and cognitive impairment. Sex-stratification was employed because the association between IGF-I and health was noted to be sex-specific in some studies [7, 19]. MMSE scores were dichotomized as described above in order to integrate into a single variable the scores that define cognitive impairment based on the regular and the blind MMSE. Furthermore, dichotomizing the MMSE into groups with and without cognitive impairment allows the results to be more clinically applicable. The models were adjusted for potential confounders of cognition and IGF-I level, which included age; history of diabetes mellitus, hypertension, cancer (excluding non-melanoma skin cancer) and cardiovascular disease (defined as a history of stroke, myocardial infarction, coronary artery bypass graft surgery, or angioplasty); depression as suggested by the GDS; education years; history of tobacco use; history of alcohol use; and IGFBP-3 level. The model in males could not sustain adjustment for all the potential covariates due to the small number of subjects. Goodness of fit for the model was assessed with the Hosmer and Lame show goodness of fit test. Sex-specific tertiles of IGF-I were created based on the study sample with available MMSE results. Known carriers of mutations in the IGF-I receptor gene (*IGF-IR*) that cause partial IGF-I resistance were excluded from this analysis because they manifest higher IGF-I levels in the setting of decreased IGF-I action [5, 6]. Results for the logistic regression models are reported as odds ratios (OR) with 95% confidence intervals (CI). A p-value <0.05 is considered statistically significant.

## References

[1] Zhu CW, Sano M, Ferris SH, Whitehouse PJ, Patterson MB, Aisen PS (2013). Health-related resource use and costs in elderly adults with and without mild cognitive impairment. J Am Geriatr Soc.

[2] Andersen SL, Sebastiani P, Dworkis DA, Feldman L, Perls TT (2012). Health span approximates life span among many supercentenarians: compression of morbidity at the approximate limit of life span. J Gerontol A Biol Sci Med Sci.

[3] Sebastiani P, Sun FX, Andersen SL, Lee JH, Wojczynski MK, Sanders JL, Yashin A, Newman AB, Perls TT (2013). Families Enriched for Exceptional Longevity also have Increased Health-Span: Findings from the Long Life Family Study. Front Public Health.

[4] Ismail K, Nussbaum L, Sebastiani P, Andersen S, Perls T, Barzilai N, Milman S (2016). Compression of Morbidity Is Observed Across Cohorts with Exceptional Longevity. J Am Geriatr Soc.

[5] Suh Y, Atzmon G, Cho MO, Hwang D, Liu B, Leahy DJ, Barzilai N, Cohen P (2008). Functionally significant insulin-like growth factor I receptor mutations in centenarians. Proc Natl Acad Sci USA.

[6] Tazearslan C, Huang J, Barzilai N, Suh Y (2011). Impaired IGF1R signaling in cells expressing longevity-associated human IGF1R alleles. Aging Cell.

[7] Milman S, Atzmon G, Huffman DM, Wan J, Crandall JP, Cohen P, Barzilai N (2014). Low insulin-like growth factor-1 level predicts survival in humans with exceptional longevity. Aging Cell.

[8] van der Spoel E, Rozing MP, Houwing-Duistermaat JJ, Slagboom PE, Beekman M, de Craen AJ, Westendorp RG, van Heemst D (2015). Association analysis of insulin-like growth factor-1 axis parameters with survival and functional status in nonagenarians of the Leiden Longevity Study. Aging (Albany NY).

[9] Brown-Borg HM, Borg KE, Meliska CJ, Bartke A (1996). Dwarf mice and the ageing process. Nature.

[10] Ikeno Y, Bronson RT, Hubbard GB, Lee S, Bartke A (2003). Delayed occurrence of fatal neoplastic diseases in ames dwarf mice: correlation to extended longevity. J Gerontol A Biol Sci Med Sci.

[11] Kenyon C, Chang J, Gensch E, Rudner A, Tabtiang R (1993). A C. elegans mutant that lives twice as long as wild type. Nature.

[12] Guevara-Aguirre J, Balasubramanian P, Guevara-Aguirre M, Wei M, Madia F, Cheng CW, Hwang D, Martin-Montalvo A, Saavedra J, Ingles S, de Cabo R, Cohen P, Longo VD (2011). Growth hormone receptor deficiency is associated with a major reduction in pro-aging signaling, cancer, and diabetes in humans. Sci Transl Med.

[13] Renehan AG, Zwahlen M, Minder C, O'Dwyer ST, Shalet SM, Egger M (2004). Insulin-like growth factor (IGF)-I, IGF binding protein-3, and cancer risk: systematic review and meta-regression analysis. Lancet.

[14] Milman S, Huffman DM, Barzilai N (2016). The Somatotropic Axis in Human Aging: Framework for the Current State of Knowledge and Future Research. Cell Metab.

[15] West DW, Phillips SM (2010). Anabolic processes in human skeletal muscle: restoring the identities of growth hormone and testosterone. Phys Sportsmed.

[16] West DW, Burd NA, Tang JE, Moore DR, Staples AW, Holwerda AM, Baker SK, Phillips SM (2010). Elevations in ostensibly anabolic hormones with resistance exercise enhance neither training-induced muscle hypertrophy nor strength of the elbow flexors. J Appl Physiol (1985).

[17] Doi T, Shimada H, Makizako H, Tsutsumimoto K, Hotta R, Nakakubo S, Suzuki T (2015). Association of insulin-like growth factor-1 with mild cognitive impairment and slow gait speed. Neurobiol Aging.

[18] Okereke O, Kang JH, Ma J, Hankinson SE, Pollak MN, Grodstein F (2007). Plasma IGF-I levels and cognitive performance in older women. Neurobiol Aging.

[19] Al-Delaimy WK, von Muhlen D, Barrett-Connor E (2009). Insulinlike growth factor-1, insulinlike growth factor binding protein-1, and cognitive function in older men and women. J Am Geriatr Soc.

[20] Tumati S, Burger H, Martens S, van der Schouw YT, Aleman A (2016). Association between Cognition and Serum Insulin-Like Growth Factor-1 in Middle-Aged & Older Men: An 8 Year Follow-Up Study. PLoS One.

[21] Faulkner JA, Larkin LM, Claflin DR, Brooks SV (2007). Age-related changes in the structure and function of skeletal muscles. Clin Exp Pharmacol Physiol.

[22] Rudman D, Kutner MH, Rogers CM, Lubin MF, Fleming GA, Bain RP (1981). Impaired growth hormone secretion in the adult population: relation to age and adiposity. J Clin Invest.

[23] Friedlander AL, Butterfield GE, Moynihan S, Grillo J, Pollack M, Holloway L, Friedman L, Yesavage J, Matthias D, Lee S, Marcus R, Hoffman AR (2001). One year of insulin-like growth factor I treatment does not affect bone density, body composition, or psychological measures in postmenopausal women. J Clin Endocrinol Metab.

[24] Rudman D, Feller AG, Nagraj HS, Gergans GA, Lalitha PY, Goldberg AF, Schlenker RA, Cohn L, Rudman IW, Mattson DE (1990). Effects of human growth hormone in men over 60 years old. N Engl J Med.

[25] Vitale G, Brugts MP, Ogliari G, Castaldi D, Fatti LM, Varewijck AJ, Lamberts SW, Monti D, Bucci L, Cevenini E, Cavagnini F, Franceschi C, Hofland LJ (2012). Low circulating IGF-I bioactivity is associated with human longevity: findings in centenarians' offspring. Aging (Albany NY).

[26] van Heemst D, Beekman M, Mooijaart SP, Heijmans BT, Brandt BW, Zwaan BJ, Slagboom PE, Westendorp RG (2005). Reduced insulin/IGF-1 signalling and human longevity. Aging Cell.

[27] Yamamoto H, Sohmiya M, Oka N, Kato Y (1991). Effects of aging and sex on plasma insulin-like growth factor I (IGF-I) levels in normal adults. Acta Endocrinol (Copenh).

[28] Aleman A, Torres-Alemán I (2009). Circulating insulin-like growth factor I and cognitive function: neuro-modulation throughout the lifespan. Prog Neurobiol.

[29] Ashpole NM, Sanders JE, Hodges EL, Yan H, Sonntag WE (2015). Growth hormone, insulin-like growth factor-1 and the aging brain. Exp Gerontol.

[30] Bondy CA, Cheng CM (2004). Signaling by insulin-like growth factor 1 in brain. Eur J Pharmacol.

[31] Pan W, Yu Y, Cain CM, Nyberg F, Couraud PO, Kastin AJ (2005). Permeation of growth hormone across the blood-brain barrier. Endocrinology.

[32] Reinhardt RR, Bondy CA (1994). Insulin-like growth factors cross the blood-brain barrier. Endocrinology.

[33] Kranzler JH, Rosenbloom AL, Martinez V, Guevara-Aguirre J (1998). Normal intelligence with severe insulin-like growth factor I deficiency due to growth hormone receptor deficiency: a controlled study in a genetically homogeneous population. J Clin Endocrinol Metab.

[34] Netchine I, Azzi S, Le Bouc Y, Savage MO (2011). IGF1 molecular anomalies demonstrate its critical role in fetal, postnatal growth and brain development. Best Pract Res Clin Endocrinol Metab.

[35] Han VK, Lund PK, Lee DC, D'Ercole AJ (1988). Expression of somatomedin/insulin-like growth factor messenger ribonucleic acids in the human fetus: identification, characterization, and tissue distribution. J Clin Endocrinol Metab.

[36] Sun LY, Al-Regaiey K, Masternak MM, Wang J, Bartke A (2005). Local expression of GH and IGF-1 in the hippocampus of GH-deficient long-lived mice. Neurobiol Aging.

[37] Matheny RW, Merritt E, Zannikos SV, Farrar RP, Adamo ML (2009). Serum IGF-I-deficiency does not prevent compensatory skeletal muscle hypertrophy in resistance exercise. Exp Biol Med (Maywood).

[38] Spangenburg EE, Le Roith D, Ward CW, Bodine SC (2008). A functional insulin-like growth factor receptor is not necessary for load-induced skeletal muscle hypertrophy. J Physiol.

[39] West DW, Burd NA, Staples AW, Phillips SM (2010). Human exercise-mediated skeletal muscle hypertrophy is an intrinsic process. Int J Biochem Cell Biol.

[40] Baker J, Liu JP, Robertson EJ, Efstratiadis A (1993). Role of insulin-like growth factors in embryonic and postnatal growth. Cell.

[41] Liu JP, Baker J, Perkins AS, Robertson EJ, Efstratiadis A (1993). Mice carrying null mutations of the genes encoding insulin-like growth factor I (Igf-1) and type 1 IGF receptor (Igf1r). Cell.

[42] Rajpathak SN, Liu Y, Ben-David O, Reddy S, Atzmon G, Crandall J, Barzilai N (2011). Lifestyle factors of people with exceptional longevity. J Am Geriatr Soc.

[43] Atzmon G, Schechter C, Greiner W, Davidson D, Rennert G, Barzilai N (2004). Clinical phenotype of families with longevity. J Am Geriatr Soc.

[44] Folstein MF, Folstein SE, McHugh PR (1975). “Mini-mental state”. A practical method for grading the cognitive state of patients for the clinician. J Psychiatr Res.

[45] Stuss DT, Meiran N, Guzman DA, Lafleche G, Willmer J (1996). Do long tests yield a more accurate diagnosis of dementia than short tests? A comparison of 5 neuropsychological tests. Arch Neurol.

[46] Busse A, Sonntag A, Bischkopf J, Matschinger H, Angermeyer MC (2002). Adaptation of dementia screening for vision-impaired older persons: administration of the Mini-Mental State Examination (MMSE). J Clin Epidemiol.

[47] Reischies FM, Geiselmann B (1997). Age-related cognitive decline and vision impairment affecting the detection of dementia syndrome in old age. Br J Psychiatry.

[48] Atzmon G, Gabriely I, Greiner W, Davidson D, Schechter C, Barzilai N (2002). Plasma HDL levels highly correlate with cognitive function in exceptional longevity. J Gerontol A Biol Sci Med Sci.

[49] Kato K, Zweig R, Schechter CB, Verghese J, Barzilai N, Atzmon G (2013). Personality, self-rated health, and cognition in centenarians: do personality and self-rated health relate to cognitive function in advanced age?. Aging (Albany NY).

[50] Milman S, Schulder-Katz M, Deluty J, Zimmerman ME, Crandall JP, Barzilai N, Melamed ML, Atzmon G (2014). Individuals with exceptional longevity manifest a delayed association between vitamin D insufficiency and cognitive impairment. J Am Geriatr Soc.

[51] Almeida OP, Almeida SA (1999). Short versions of the geriatric depression scale: a study of their validity for the diagnosis of a major depressive episode according to ICD-10 and DSM-IV. Int J Geriatr Psychiatry.

[52] Janssen I, Heymsfield SB, Baumgartner RN, Ross R (2000). Estimation of skeletal muscle mass by bioelectrical impedance analysis. J Appl Physiol (1985).

[53] Janssen I, Baumgartner RN, Ross R, Rosenberg IH, Roubenoff R (2004). Skeletal muscle cutpoints associated with elevated physical disability risk in older men and women. Am J Epidemiol.

[54] Cruz-Jentoft AJ, Baeyens JP, Bauer JM, Boirie Y, Cederholm T, Landi F, Martin FC, Michel JP, Rolland Y, Schneider SM, Topinková E, Vandewoude M, Zamboni M, European Working Group on Sarcopenia in Older People (2010). Sarcopenia: European consensus on definition and diagnosis: Report of the European Working Group on Sarcopenia in Older People. Age Ageing.

[55] Oh-Park M, Holtzer R, Xue X, Verghese J (2010). Conventional and robust quantitative gait norms in community-dwelling older adults. J Am Geriatr Soc.

[56] Fried LP, Tangen CM, Walston J, Newman AB, Hirsch C, Gottdiener J, Seeman T, Tracy R, Kop WJ, Burke G, McBurnie MA, Cardiovascular Health Study Collaborative Research Group (2001). Frailty in older adults: evidence for a phenotype. J Gerontol A Biol Sci Med Sci.

[57] Ayers E, Barzilai N, Crandall JP, Milman S, Verghese J (2014). Association of exceptional parental longevity and physical function in aging. Age (Dordr).

[58] Guralnik JM, Ferrucci L, Pieper CF, Leveille SG, Markides KS, Ostir GV, Studenski S, Berkman LF, Wallace RB (2000). Lower extremity function and subsequent disability: consistency across studies, predictive models, and value of gait speed alone compared with the short physical performance battery. J Gerontol A Biol Sci Med Sci.

